# Elevated glucose increases methicillin-resistant Staphylococcus aureus antibiotic tolerance in a cystic fibrosis airway epithelial cell infection model

**DOI:** 10.21203/rs.3.rs-5938603/v1

**Published:** 2025-02-17

**Authors:** Emily M. Hughes, Meghan J. Hirsch, Joshua T. Huffines, Stefanie Krick, Megan R. Kiedrowski

**Affiliations:** University of Alabama at Birmingham; University of Alabama at Birmingham; University of Alabama at Birmingham; University of Alabama at Birmingham; University of Alabama at Birmingham

## Abstract

**Background:**

In a healthy lung, the airway epithelium regulates glucose transport to maintain low glucose concentrations in the airway surface liquid (ASL). However, hyperglycemia and chronic lung diseases, such as cystic fibrosis (CF), can result in increased glucose in bronchial aspirates. People with CF are also at increased risk of lung infections caused by bacterial pathogens, including methicillin-resistant *Staphylococcus* aureus. Yet, it is not known how increased airway glucose availability affects bacteria in chronic CF lung infections or impacts treatment outcomes.

**Methods:**

To model the CF airways, we cultured immortalized CF (CFBE41o-) and non-CF (16HBE) human bronchial epithelial cells at air liquid interface (ALI). Glucose concentrations in the basolateral media were maintained at 5.5 mM or 12.5 mM, to mimic a normal and hyperglycemic milieu respectively. 2-deoxyglucose was added to high glucose culture media to restrict glucose availability. We collected ASL, basolateral media, and RNA from ALI cultures to assess the effects of elevated glucose. We also inoculated *S. aureus* onto the apical surface of normal or high glucose ALI cultures and observed the results of antibiotic treatment post-inoculation. *S. aureus* growth was measured by enumerating viable colony forming units (CFU) and with fluorescence microscopy. The effects of elevated glucose on *in vitro* growth and antibiotic treatment were also evaluated in standard bacterial culture medium and synthetic CF medium (SCFM).

**Results:**

We found that glucose concentrations in the ASL of ALI cultures maintained in normal or high glucose mimicked levels measured in breath condensate assays from people with CF and hyperglycemia. Additionally, we found hyperglycemia increased *S. aureus* aggregation and antibiotic resistance during infection of cells maintained in high glucose compared to normal glucose conditions. Heightened antibiotic tolerance or resistance as not observed during *in vitro* growth with elevated glucose. Limiting glucose with 2-deoxyglucose both decreased aggregation and reduced antibiotic resistance back to levels comparable to non-hyperglycemic conditions.

**Conclusions:**

These data indicate hyperglycemia alters *S. aureus* growth during infection and may reduce efficacy of antibiotic treatment. Glucose restriction is a potential option that could be explored to limit bacterial growth and improve treatment outcomes in chronic airway infections.

## BACKGROUND

Cystic fibrosis (CF) is a rare genetic disease, which affects multiple organs in the body including the lungs. Mutations in the cystic fibrosis transmembrane conductance regulator (CFTR) gene result in improper ion transport resulting in decreased mucociliary clearance and accumulation of thick and sticky mucus on epithelial surfaces (1, 2). In the lungs, abnormal mucus traps bacterial pathogens and provides an environment that harbors persistent infection because of mucus that cannot be properly cleared from the lungs, resulting in chronic inflammation (2,3). In CF, the pancreas is also affected by mucus build up that causes cellular damage and scarring, preventing insulin from being made while also creating a physical barrier preventing insulin from being properly secreted (4,5). Additionally, there is often pancreatic remodeling leading to the loss of beta cells reducing the amount of insulin being produced. This results in onset of CF related diabetes (CFRD) in approximately 30% of the US CF population (6, 7).

*Staphylococcus aureus* is the most common pathogen isolated from the CF lung. Even with the introduction of highly effective modulator therapy (HEMT), people with CF (pwCF) still have very high rates of positive *S. aureus* cultures, with around 60% of the US CF population having at least one *S. aureus* positive culture in 2023 (7–9). Methicillin resistant *S. aureus* (MRSA) is a continued health concern in chronic respiratory diseases including CF, due to antibiotic resistance (10,11). Studies in non-CF populations have shown that having diabetes increases the risk of health care-associated pneumonia caused by MRSA, while other studies have shown that ICU patients that are intubated and have ≥ 1 mM of glucose in their bronchial aspirates had significantly more staphylococcus species present, including significantly more MRSA present (12–14). This correlation is of particular importance to pwCF because HEMT has not reduced the rates of CFRD, and insulin is currently the only treatment option for maintaining blood glucose levels (15–17). CFRD is a significant risk factor for developing persistent MRSA infections and MRSA and CFRD have been shown to lead to worse outcomes than either factor alone (18, 19).

It has been hypothesized that glucose restriction is one factor that helps to keep the airway sterile by limiting nutritional availability for microbes that colonize the respiratory tract or to prevent the invasion by pathogens such as *S. aureus*. A healthy lung epithelium tightly regulates glucose homeostasis, keeping free glucose concentrations well below 1 mM in the airway (20, 21). Hyperglycemia disrupts this homeostasis, increasing airway glucose concentrations up to 1.89 mM. Chronic lung diseases, like CF, further disrupt glucose homeostasis, with pwCF found to have up to 3.13 mM glucose in bronchial aspirates (21–23). CFRD causes a further increase, resulting in up to 6.07 mM of available glucose in the lung (21).

Despite the correlation between diabetes and *S. aureus* lung infections, very few studies have examined how the diabetic lung environment and increase in airway glucose availability impact *S. aureus* infections or how these conditions might affect antibiotic treatment outcomes. In this study, we use human bronchial epithelial cells cultured in elevated glucose conditions to mimic the normal and hyperglycemic lung environment. Using this model, we found that the hyperglycemic lung environment significantly decreases *S. aureus* response to antibiotic treatment, and limiting glucose availability at the airway surface can reverse this effect.

## MATERIALS AND METHODS

### Bacterial strains and growth conditions

*A Staphylococcus aureus* USA100 strain representative of MRSA isolates commonly observed in CF was used for these studies (24). *S. aureus* with or without plasmid pCM29 encoding green fluorescent protein (GFP) (25) was cultured overnight at 37°C with shaking in tryptic soy broth (TSB; BD Bioscience). Overnight cultures were inoculated using a single colony grown on TSB with 1.5% agar (TSA, BD Bioscience) at 37°C. Bacterial growth curves were done using TSB without dextrose (BD Bioscience) supplemented with glucose (Gibco) and/or 2-deoxyglucose (2DG, EMD Millipore Sigma) to achieve final concentrations. Approximately 1 × 10^6^ bacteria were inoculated per well in 96 well plates. Growth curves were run for 30 hr total in a Tecan Spark automated multimode microplate reader. If antibiotic was added after 6 hr of growth, the microplate reader was paused, and rifampicin (Fisher BioReagents) was added to equal a final concentration of 35 μg/mL per well.

A modified synthetic cystic fibrosis medium (SCFM) was made according to Palmer et al with glucose added to achieve 0-, 1-, 3-, 5-, or 10 mM final concentrations (26). When antibiotic was added at time of inoculation, bacterial colony forming units (CFUs) were determined at time of inoculum and at the assay end point. Bacteria were plated to both antibiotic-free TSA plates and to TSA with rifampicin (10 μg/mL) to determine total bacterial burden and the burden of rifampicin-resistant bacteria. For assays where antibiotic was added after 6 hr of growth, CFUs were determined for the inoculum, at time of antibiotic addition (6 hr), and endpoint (24 hr) CFUs were measured by plating to TSA plates with and without rifampicin.

### Cell culture

#### Maintenance and ALI culture

Both non-CF (16HBE) and CF (CFBE41o-) immortalized human bronchial epithelial cells were maintained as previously described (24) in minimal essential medium (MEM; Gibco) supplemented with 10% fetal bovine serum (FBS; Gibco) and pen-strep. Briefly, air-liquid interface cultures were established by seeding cells on transwell filters pre-coated with vitrogen plating media (VPM). After one week, media was removed from the apical side of the transwell, and cells were cultured at the air-liquid interface for at least one week before use in downstream assays. Cells were cultured at normal glucose (5.5 mM) until 24 or 48 hr before use. At that time, ALI cultures were washed with MEM lacking serum and phenol red (Gibco) to remove any residual antibiotics. ALI cultures were then fed with antibiotic free media with normal glucose (5.5 mM), hyperglycemic glucose (12.5 mM), or 2DG (7 mM 2DG + 5.5 mM glucose). Hyperglycemic media was made by adding glucose to the base MEM cell culture media. MEM containing 2DG (EMD Millipore Sigma) was made by adding 2DG to base MEM for a final concentration of 7 mM 2DG and 5.5 mM glucose.

For ASL collection, 24 hours after media change, 250 μl of clear, FBS-free MEM was added to the apical side of ALI cultures grown in 12 mm transwell inserts and incubated for 24 hrs. The apical media was then collected, and glucose concentrations were determined using a glucose assay kit (Abcam). Transepithelial electrical resistance (TEER) was also measured after 6 and 24 hr post-addition of clear MEM to ALI cultures using an EVOM2 epithelial Volt/Ohm meter (World Precision Instruments). Additionally, at the 24 hr timepoint, basolateral media was collected to measure lactate dehydrogenase release (LDH, Promega). Further, some ALI samples were washed twice with ice cold PBS++ (Gibco) and stored at −80°C for subsequent RNA extraction.

### Co-culture infection assay

24 hr after changing basolateral media of ALI cultures to different glucose conditions, as described above, ALI cultures were infected with 1×10^6^ colony forming units (CFUs) of live bacteria. After 1 hr, media containing any unattached bacteria was removed. CFUs were determined by addition of 0.1% triton (Bio-rad) to infected ALI samples, scraping total cells and bacteria, and plating serial dilutions on plain TSA or TSA with rifampicin (10 μg/mL) to enumerate total and antibiotic-resistant *S. aureus* populations (27). At 6 hr post-inoculation, some infected ALI cultures were harvested for CFUs or imaging. At 6 hr, some ALI cultures were treated with antibiotic (rifampicin 35 μg/mL) or vehicle control (MEM) added to the apical surface, and infection was allowed to proceed for an additional 24 hr. After 24 hr of treatment, final endpoint CFUs were determined as described above.

### Fluorescence microscopy and biomass measurements

After 6 hr of co-infection described above, ALI cultures were fixed in 4% paraformaldehyde (PFA, Electron Microscopy Sciences). After overnight fixation, transwell filters were washed twice with PBS (Gibco) then stained with Hoechst 33342 stain (Invitrogen). Filters were then cut out from transwell inserts using a razor blade and mounted on a microscope slide with Prolong Gold (Invitrogen). After drying, the filters were imaged on a widefield Ti Eclipse widefield fluorescence microscope (Nikon). After imaging, quantification of biofilms was done using the Nikon NIS-Elements AR software package (Version 5.42.02 Build 1801). Volume measurements were obtained for each image stack after automatic thresholding was performed in NIS-Elements AR. The NIS-Elements object count function was used to determine the number and area of bacterial aggregates. Data analysis for bacterial aggregates was performed using RStudio version 2024.09.0 Build 375 “Cranberry Hibiscus” Release (Posit Software). Aggregates with an area value less than 5 μm in size were excluded to eliminate noise and single cells from data analysis (28, 29). Images shown and biomass measurements are representative of at least three independent experiments with at least five individual fields of view measured for each sample.

### RNA extraction and cytokine measurements

After treatments with glucose and/or *S. aureus*, total RNA was isolated, and RT-qPCR was run as previously described (30). Briefly, RNA was isolated using a GeneJET Purification Kit (ThermoScientific). RNA concentrations were assessed using a Nanodrop and cDNA was synthesized using a Maxima H Minus cDNA Synthesis Master Mix (ThermoFisher). RT-qPCR was performed on an Applied Biosystems StepOnePlus using TaqMan primers Interleukin 1-β (Hs01555410_m1, IL-1β), Interleukin-6 (Hs00174131_m1, IL-6), and Interleukin-8 (Hs00174103_m1, CXCL8) and reference gene glyceraldehyde 3-phosphate dehydrogenase (GAPDH). Relative difference in transcript levels was calculated using the ΔΔCt method with GAPDH as a reference gene.

Additionally, secreted IL-1β, IL-6, and IL-8 protein levels were measured after treatments with glucose and/or *S. aureus* by ELISAs (Invitrogen). These were performed according to manufacturer’s protocol with the following assay sensitivities: human IL-1β (0.16–10pg/mL), human IL-6 (2–200 pg/mL), and human IL-8 ELISA (2–250 pg/mL).

### Statistical analysis

Statistical analyses were performed with Graph Pad Prism version 10.4.0 software (GraphPad by Dotmatics). Two-way analysis of variance (ANOVA) was determined as appropriate to measure statistical differences between cell types and glucose conditions. Tukey post hoc testing was performed on multiple comparisons. P values were considered significant if less than 0.05.

## RESULTS

### A hyperglycemic air-liquid interface cell culture model accurately replicates glucose levels measured in the human lung.

**Figure 1:**
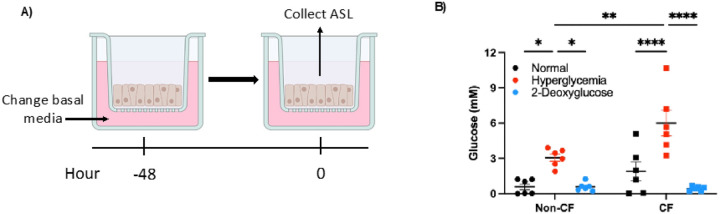
Hyperglycemic conditions alter glucose concentrations in the airway surface liquid (ASL). A) Cells at air liquid interface (ALI) were switched to different glucose media 48 hr prior to experiments. B) ASL glucose measurements from both non-CF (circles) and CF (squares) cells. Data reported as mean ± SEM. *P < 0.05, **P < 0.01, ****P < 0.0001

To determine the impact glucose has on *S. aureus*, we first characterized our cell culture model using cells cultured at air-liquid interface (ALI). Using the physiological relevant glucose conditions of 5.5 mM to represent normal blood glucose levels and 12.5 mM to represent a hyperglycemic blood glucose level. After establishing our cell culture conditions, we then measured the amount of glucose found in the airway surface liquid (ASL) (Fig. 1A). We found that our model closely replicates ASL glucose concentrations measured in clinical samples (Fig. 1B) (21). We then wanted to determine if we could limit the glucose in the ASL. To accomplish this, we used a competitive inhibitor, 2-Deoxyglucose (2DG) (31). Using this, we found that it significantly lowered glucose levels in the ASL (Fig. 1B). To further characterize our model, and because 2DG inhibits glycolysis, which can cause cell death, we verified the cell monolayer was still intact using transepithelial electrical resistance (TEER) (Supplemental Fig. 1A). This also confirmed that the increase in ASL glucose is not due to a disruption in tight junction integrity.

We also determined no significant increase in lactate dehydrogenase (LDH), indicating that there is not an increase in cellular cytotoxicity (Supplemental Fig. 1B). Together, from these experiments we can conclude that our model accurately represents the glucose conditions found in the lung and that hyperglycemia and the competitive inhibitor 2DG does not have a negative impact on our model.

### Short-term high glucose ALI culture of immortalized bronchial epithelial cells does not alter inflammatory cytokine levels.

**Figure 2:**
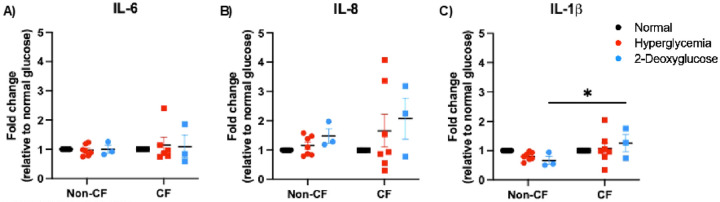
Hyperglycemia does not meaningfully impact pro-inflammatory cytokine levels. A) IL-6 transcript levels B) IL-8 transcript levels & C) IL-1β transcript levels. Data reported as mean ± SEM. *P < 0.05

Because individuals with hyperglycemia and CF are known to have increased systemic inflammation, we wanted to confirm if glucose levels affect pro-inflammation cytokine expression in this model (32–34). Therefore, we measured the expression of common pro-inflammatory cytokines, IL-6, IL-8 & IL-1β via RT qPCR (Fig. 2A–C). We observed that hyperglycemia did not significantly elevate inflammatory cytokines in either non-CF or CF cells compared to normal controls. Only IL-β levels were found to be significantly increased in 2DG treated CF cells and the expected differences between cell types were maintained (Fig. 2C and Supplemental Fig. 2). These data demonstrate that hyperglycemia alone is not significantly increasing inflammation in our model.

### S. aureus aggregate size is increased in CF hyperglycemic conditions.

**Figure 3:**
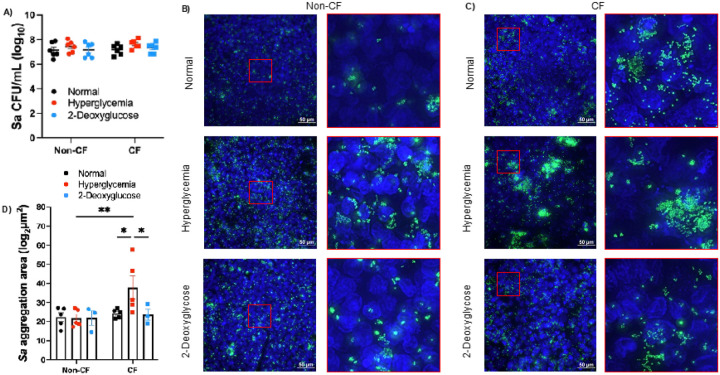
The hyperglycemic airway environment alters *S. aureus* aggregation. *S. aureus* grown on non-CF or CF cells for 6 hrs before experiments. A) *S. aureus* (Sa) bacterial burden. B) Widefield microscopy images of *S. aureus* (green) on human bronchial cells (blue) non-CF or C) CF. D) Quantification of aggregation area for aggregates over 5 μm. Data reported as mean ± SEM. *P < 0.05, **P < 0.01

After confirming our model replicates clinical data and that we can control ASL glucose levels with no adverse effects to the epithelial cell monolayer, we then infected our ALI model with *S. aureus* USA100, a hospital acquired strain of MRSA. We determined that there is no difference in *S. aureus* burden after 6 hr on non-CF and CF cells cultured in normal or hyperglycemic conditions, and 2DG treatment does not significantly affect total bacterial burden (Fig. 3A). However, imaging revealed a significant increase in the number of bacterial aggregates measuring over 5 μm in CF hyperglycemic conditions but not in non-CF hyperglycemic conditions (Fig. 3D). This cut-off was chosen as it has been previously determined that this is the average size of bacterial isolates from the airways of people with CF (28, 29). The addition of 2DG reduced aggregate size back to normal glucose conditions (Fig. 3B&C).

### Antibiotic resistance is increased in ALI co-culture in hyperglycemic conditions.

**Figure 4:**
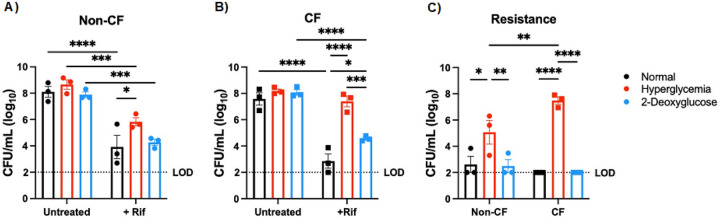
High glucose increases rifampicin resistance in *S. aureus* when co-cultured with human bronchial cells. CFUs from USA100 co-cultured with cells with or without rifampicin treatment. A) Non-CF cells. B) CF cells. C) Resistance from both non-CF (circles) and CF (squares) cells. Horizontal line indicates limit of detection (LOD). Data reported as mean ± SEM. *P < 0.05, **P < 0.01, ***P < 0.001, ****P < 0.0001

After observing that elevated glucose increases bacterial aggregation in our ALI model, we next wanted to determine the effects of hyperglycemia on the outcome of antibiotic treatment. To mimic an antibiotic intervention, we infected normal or hyperglycemic ALI cultures with *S. aureus* for 6 hr to allow biofilms to establish and mimic an existing infection, before adding rifampicin (RIF) and allowing the infection to proceed for an additional 24 hr. We found that in the non-CF cells, antibiotic treatment significantly reduced *S. aureus* burden regardless of glucose condition (Fig. 4A). Similarly, with CF cells, we found that in normal glucose conditions, RIF reduced bacterial burden to almost undetectable levels. However, culturing cells in hyperglycemic conditions completely negated the effects of antibiotic treatment, with no difference in total *S. aureus* burden between antibiotic treated and untreated conditions (Fig. 4B). To determine if RIF resistance was developing in *S. aureus*, leading to the differences we observed, we also measured the number of resistant bacteria at the final timepoint by enumerating colonies that grew on culture media containing rifampicin. We found there were significantly more resistant bacteria in hyperglycemic samples treated with antibiotic than in the normal conditions also with antibiotic treatment (Fig. 4C). Surprisingly, this was not dependent on cell type. The bacteria on the non-CF cells developed resistance as frequently when exposed to the antibiotic as on the CF cells despite RIF still being effective at reducing overall bacterial burden on the non-CF cells. Bacterial resistance was not observed in inoculums or at the 6 hr time point in either condition (data not shown). To confirm that this was a glucose-dependent response, we again utilized 2DG to limit ASL glucose. 2DG treatment significantly decreased the number of resistant *S. aureus* colonies, and the total burden of RIF-resistant *S. aureus* resembled the burden observed in normal glucose conditions for both cell types (Fig. 4A, B, & C).

### Elevating glucose in in vitro culture does not result in increased antibiotic tolerance or development of resistance.

**Figure 5:**
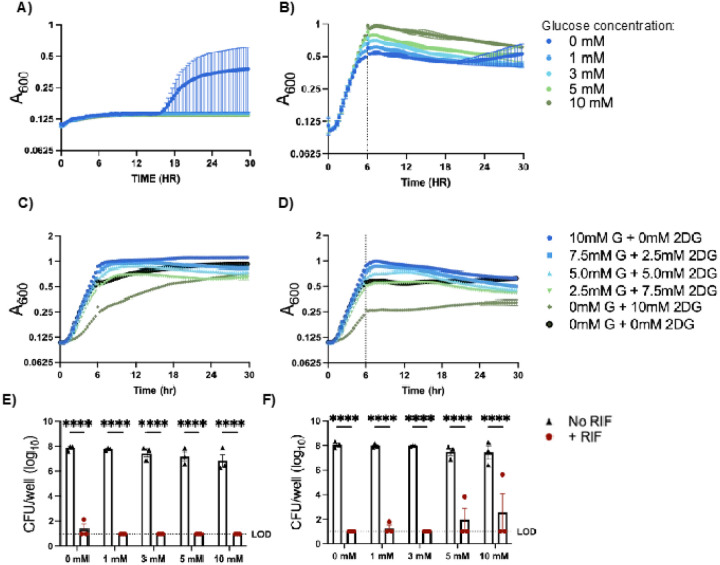
Rifampicin is effective at killing bacteria in broth. USA100 grown in rich media or defined media with increasing amounts of glucose. Rifampicin added at the same time or after 6 hr of growth. A) Growth curve in rich media with rifampicin added at the same time. B) Growth curve with rich media with rifampicin added after 6 hr of growth. Vertical dashed indicated addition of antibiotic. C) Growth of USA100 in rich media with glucose and 2-deoxyglucose (2DG) without rifampicin. D) Growth of USA00 in rich media with glucose and 2DG with rifampicin added after 6 hrs of growth. Vertical dashed line indicates addition of antibiotic. E) CFUs from defined media with rifampicin added at the same time. Horizontal dashed line indicated limit of detection (LOD). F) CFUs from defined media with rifampicin added after 6 hr of growth. Horizontal dashed line indicated limit of detection (LOD). Data reported as mean ± SEM. ****P < 0.0001

After seeing the differences in bacterial aggregation and antibiotic tolerance resulting from elevated glucose in our cell culture model, we next asked if these effects could be mimicked in broth culture in the absence of airway epithelial cells. To do this, we utilized a rich culture media commonly used for growth of *S. aureus*, tryptic soy broth (TSB), and defined media replicating the nutrients available in the CF lung environment, synthetic CF media (SCFM) (26). We prepared base media lacking glucose and added defined amounts of glucose to achieve final concentrations that mimicked levels measured in ASL of non-CF and CF cells cultured in normal or hyperglycemic conditions. Using this range of glucose concentrations, we found that in both rich media and defined media, the addition of RIF, was able to prevent growth of *S. aureus* when added concurrently with the bacterial inoculum (Fig. 5A & 5E). However, one biological replicate grown in 0 mM glucose media developed resistance to RIF in TSB and SCFM (Fig. 5A and Supplemental Fig. 3A). This was not observed in other glucose concentrations and was most likely due the fact that spontaneous RIF resistance can occur (35). We next grew *S. aureus* for 6 hr before adding RIF, to mimic an existing infection as tested in the ALI co-infections. In TSB, we observed similar results as with ALI infections, where RIF was effective at killing *S. aureus*. Here, we also observed that one replicate developed resistance (Fig. 5B). RIF was also effective at killing *S. aureus* in SCFM, however, here the 1mM, 5mM, & 10mM conditions each had one replicate develop resistance (Supplemental Fig. 3B).

Additionally, culturing *S. aureus* in media containing glucose and 2DG did not adversely affect growth (Fig. 5C) and the addition of RIF after 6 hr was still effectively reduced bacterial burden (Fig. 5D). These results indicate that allowing *S. aureus* to establish biofilm before antibiotic exposure may increase the risk of antibiotic resistance developing. Overall, we did not find that elevated glucose in either TSB or SCFM promoted RIF tolerance or the development of resistance to similar levels as observed in airway cell co-culture, suggesting airway epithelial physiology in hyperglycemic conditions may result in altered host-pathogen interactions leading to reduced antibiotic effectiveness.

## DISCUSSION

In this study, we established an air-liquid interface cell culture hyperglycemia model for the CF airway that replicates the elevated glucose levels previously been reported from the lung of those with diabetes and CF (21). We found no adverse effects to the cells due to exposure to elevated glucose in growth media and no increase in inflammatory cytokines between non-CF and CF cell types. However, we did find glucose-dependent differences, with CF cells showing elevated baseline ASL glucose concentrations that further increased upon hyperglycemic culture. Other studies have cultured cells in hyperglycemic conditions for one to three weeks and have showed varying results in changes to cell monolayer integrity as measured by TEER (36, 37). Additionally, Bengtson et al. found that non-CF primary bronchial epithelial cells cultured at 12.5 mM glucose for three weeks had increases in IL-6, IL-8 and IL-1β mRNA, while primary CF cells did not have the same increase in mRNA (36). Our model exposed immortalized cells to high glucose for a short time period. Testing prolonged exposure to hyperglycemic conditions may result in similar increases in inflammation as observed in these studies.

Despite not observing a difference in overall *S. aureus* burden on cells cultured in hyperglycemic conditions, we saw that *S. aureus* formed larger aggregates on CF cells cultured in high glucose than in other conditions. Persistent infections driven by the formation of biofilm-like aggregates are a hallmark of chronic CF airway infections. Yet, the increased aggregate size we observed on CF cells cultured at high glucose is surprising because it has been shown that glucose can disperse *S. aureus* biofilm through the inhibition of agr. Our results could be explained by the difference in glucose concentrations, the highest amount of glucose in our study was 10 mM, while studies showing glucose-induced dispersal were two to ten times that amount (38, 39). Additionally, it has been shown that it is not actually glucose that is causing the dispersal, but rather the low acidity environment generated by *S. aureus* utilization of glucose (40).

Our finding that elevated ASL glucose results in different outcomes to antibiotic treatment may help to explain why people with increased airway glucose are more susceptible to persistent *S. aureus* lung infections. Our hyperglycemic ALI co-infection culture model can also be used in future studies to determine how HEMT affect glucose availability at the airway epithelial interface and infection outcomes. Currently, there have been no clinical studies to determine how HEMT alters the amount of glucose found in the lungs of pwCF or how the bacteria present in the lung are changing with restoration of CFTR activity. If it is determined that HEMT does not correct elevated lung glucose levels, one potential treatment strategy could be augmenting antibiotic treatments with a glucose restriction therapy like 2-deoxyglucose, as our model showed no adverse effects with short term use and effective reduction in bacterial loads.

There are some limitations to our model, including a lack of innate immune cells which could have an altered response in hyperglycemic conditions and could exacerbate inflammatory cytokines. Studies with primary cell lines have shown increased cytokine levels in response to hyperglycemia, albeit for a much longer exposure time. The use of primary cell ALI cultures would allow us to address the question of how hyperglycemia is affecting ciliary beat and mucociliary clearance, both of which are important for clearing airway mucus and preventing infections. We also know that many infections in CF are not isolated to one species of bacteria but are polymicrobial in nature and even include viral infections. Not only do we not know if other bacteria can benefit from the increase in airway glucose, but we also do not know how their presence is affecting *S. aureus* in these elevated glucose conditions. Further, it is known that viral infections can alter the hosts metabolism, so further exploration of how virus-induced metabolic changes are altering the lung environment and if they are altering glucose availability in airway are important considerations for future studies.

## Data Availability

All data associated with this work is freely available upon publication. Reagents and model organisms generated through this work, including bacterial strains, will be made available for distribution upon publication.
